# A randomised controlled trial of azithromycin therapy in bronchiolitis obliterans syndrome (BOS) post lung transplantation

**DOI:** 10.1136/thoraxjnl-2014-205998

**Published:** 2015-02-24

**Authors:** Paul A Corris, Victoria A Ryan, Therese Small, James Lordan, Andrew J Fisher, Gerard Meachery, Gail Johnson, Chris Ward

**Affiliations:** 1 Institute of Transplantation, Freeman Hospital, Newcastle upon Tyne, UK; 2 Institute of Cellular Medicine, Newcastle upon Tyne, UK; 3 Institute of Health and Society Newcastle University, Newcastle upon Tyne, UK

**Keywords:** Lung Transplantation

## Abstract

**Background:**

We conducted a placebo-controlled trial of azithromycin therapy in bronchiolitis obliterans syndrome (BOS) post lung transplantation.

**Methods:**

We compared azithromycin (250 mg alternate days, 12 weeks) with placebo. Primary outcome was FEV_1_ change at 12 weeks.

**Results:**

48 patients were randomised; (25 azithromycin, 23 placebo). It was established, post randomisation that two did not have BOS. 46 patients were analysed as intention to treat (ITT) with 33 ‘Completers’. ITT analysis included placebo patients treated with open-label azithromycin after study withdrawal.

**Outcome:**

The ITT analysis (n=46, 177 observations) estimated mean difference in FEV_1_ between treatments (azithromycin minus placebo) was 0.035 L, with a 95% CI of −0.112 L to 0.182 L (p=0.6). Five withdrawals, who were identified at the end of the study as having been randomised to placebo (four with rapid loss in FEV_1_, one withdrawn consent) had received rescue open-label azithromycin, with improvement in subsequent FEV_1_ at 12 weeks. Study Completers showed an estimated mean difference in FEV_1_ between treatment groups (azithromycin minus placebo) of 0.278 L, with 95% CI for the mean difference: 0.170 L to 0.386 L (p=<0.001). Nine of 23 ITT patients in the azithromycin group had ≥10% gain in FEV_1_ from baseline. No patients in the placebo group had ≥10% gain in FEV_1_ from baseline while on placebo (p=0.002). Seven serious adverse events, three azithromycin, four in the placebo group, were deemed unrelated to study medication.

**Conclusions:**

Azithromycin therapy improves FEV_1_ in patients with BOS and appears superior to placebo. This study strengthens evidence for clinical practice of initiating azithromycin therapy in BOS.

**Trial registration number:**

EU-CTR, 2006-000485-36/GB.

**Key messages**

**What is the key question?**

- A number of international centres have reported a clinically significant response to azithromycin therapy in open studies of lung transplant recipients with bronchiolitis obliterans syndrome (BOS) but there are also negative studies, where azithromycin therapy was not associated with gain in lung function.

**What is the bottom line?**

- With no randomised placebo controlled studies performed or published there was a clear unmet need, met by this trial, which showed on average that azithromycin was superior to placebo treatment in our study population.

**Why read on?**

- This study outlines strengthened evidence for the clinical practice of initiating azithromycin therapy for patients who develop BOS post lung transplantation.

## Introduction

Lung transplantation can be the only life-sustaining intervention for end-stage lung disease.^1^ Good functional outcomes have been shown, with improved quality of life.^2^ Long-term survival remains limited by the development of the bronchiolitis obliterans syndrome (BOS) however.^3^

The histological lesion of BOS is obliterative bronchiolitis. This is characterised by epithelial alloimmune and non-alloimmune injury.^4^ ^5^ Deterioration in allograft function is characterised by the development of progressive, small airway narrowing, fixed airflow limitation, progressive dyspnoea and premature death.^6^ International data shows in excess of 50% of patients surviving to 5 years after transplantation develop BOS,^6^ limiting 10-year survival to around 30%.^7^

Therapeutic approaches have ranged from switching immunosuppression^6^ through to initiating cytolytic therapy. Such approaches, including the use of total lymphoid irradiation^8^ have, at best, reduced the rate of decline in graft function in BOS, with significant iatrogenic potential.^8^

In contrast, retrospective studies of macrolides, in particular low dose azithromycin, have indicated that up to 30% patients with BOS may gain lung function. A number of international centres have reported a clinical response to azithromycin therapy in around a third of patients,^9–13^ with better life expectancy.^13^ There have been negative studies, however, with no gain in lung function.^14–16^ The need for randomised controlled trial data has been highlighted,^17^ but these have not been performed.

We tested the hypothesis that azithromycin therapy is superior to placebo in patients with BOS in a randomised double blind placebo controlled study. Some results have been presented as abstracts.^18^ ^19^

## Methods

### Study design

#### Randomisation and masking

This was a single-centre randomised double-blind placebo-controlled parallel group study comparing azithromycin (250 mg on alternate days) with placebo over 12 weeks in lung transplant recipients with BOS, with study drug taken in addition to existing medication. Patients were randomly assigned to a treatment arm in a 1:1 ratio using random permuted blocks within strata. Study medication was provided by Bilcare, (Bilcare GCS Europe, Powys, UK) a commercial clinical trial supplier, independent of the manufacturers of azithromycin.

### Patient population

Patients were recruited between November 2006 and December 2010 from the Freeman Hospital.

### Withdrawal of patients from study

Patients who had a rapid and severe deterioration in lung function were withdrawn from the study (for patient details see online supplementary appendix 2). This was defined a priori as a sustained 500 mL fall in FEV_1_ from baseline, before the full 12-week course of study treatment, thought to be due to BOS. Patients could also be withdrawn based on the clinical judgement of the responsible clinician. Following withdrawal patients were treated according to the usual centre and international practice, which included the use of open-label azithromycin. The randomised treatment allocation of withdrawn patients remained concealed.

### Assessments

#### Spirometry

FEV_1_ and FVC were measured at baseline, week 4, week 8 and week 12 in the Freeman Hospital.

#### Bronchoscopy, bronchoalveolar lavage and transbronchial biopsy

Patients underwent bronchoscopy at baseline (prerandomisation) and at final visit (week 12) as previously described.^20^ Transbronchial biopsies were taken at each allograft bronchoscopy, fixed in 10% formalin, embedded in paraffin, and then stained with haematoxylin and eosin (H&E) to assess acute vascular and airway inflammation according to standard ISHLT criteria by a pathologist.^21^

### Outcome measures

The primary outcome measure was change in FEV_1_ from baseline to 12 weeks. Secondary outcome measures reported here are change in FVC from baseline and change in bronchoalveolar lavage (BAL) neutrophils.

### Study oversight

Newcastle University Clinical Trials Unit monitored the study. An independent data monitoring committee was established to assess accumulating recruitment, safety and efficacy data and to oversee the trial conduct (Statistician (chair) and two consultant respiratory physicians).

### Sample size

Estimates of SDs of differences in FEV_1_ from baseline to 12 weeks in patients with lung transplant with BOS were based on the Freeman Hospital data.^41^ A sample size of 64 patients, 32 patients per randomised group, allowed for 10% data attrition. A recruitment period of 30 months was estimated to be adequate to recruit 64 patients.

### Statistical methods

The mean difference in FEV_1_ between treatment groups was estimated using a multilevel random effects model assuming a normal error structure within and between patients.^22^ The models were fitted in MLwiN software (V.2.28).^23^ Random effects models allow appropriate estimation of the treatment effect (and associated SE) taking into account varying numbers of measurements within patients and varying time between measurements. Models were also adjusted for baseline FEV_1_ and the two randomisation stratification variables.^24^ Model assumptions were checked and analyses omitting possible outliers or influential observations were performed. The secondary outcome measure, FVC, was analysed in the same way.

BAL neutrophil counts and their change from baseline to 12 weeks were summarised and presented as median and IQR.

The intention-to-treat population (ITT) was defined as all randomised patients with BOS who received at least one dose of study drug. The per protocol population was defined as all randomised patients with BOS who followed the protocol and completed 12 weeks of study drug. Completers included all randomised patients with BOS who completed 12 weeks of study drug.

An ‘as treated’ analysis was also performed; this was a ‘post hoc’ analysis which had not been described in the statistical analysis plan and as such should be interpreted cautiously. Patients who withdrew or were withdrawn from study drug were treated with open-label azithromycin. The ‘as treated’ analysis provided an estimate of the treatment effect allowing for treatment to change over time. In this way a patient's measurement contributed to the treatment effect based on the treatment they were receiving at the time the measurement was taken and not the treatment as randomised (for extended details see online supplementary appendix 1).

## Results

### Study patients

**Figure 1:**
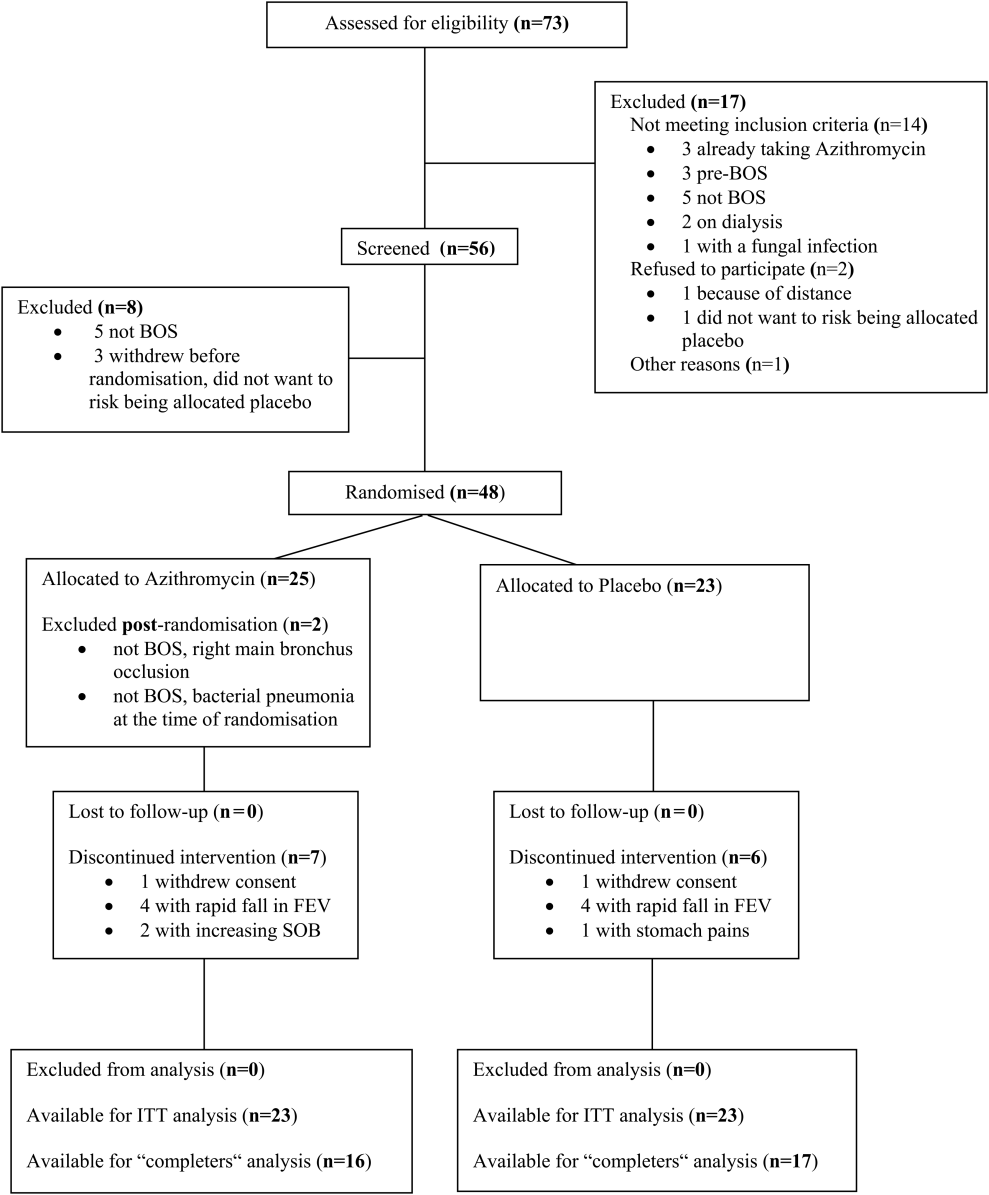
CONSORT flow chart^25^ summarising the progress of patients through the trial. BOS, bronchiolitis obliterans syndrome; ITT, intention-to-treat.

The CONSORT flow chart^25^ is presented in figure 1. Patient withdrawals are detailed in the online supplementary appendix 2 (for extended details see online supplementary appendix 1).

All 46 patients in the ITT analysis set had baseline and final visit FEV_1_ measured. Across all visits there were 177 FEV_1_ measurements: 2 patients had 5 FEV_1_ measurements, 36 patients had 4, 7 patients had 3, and 1 patient had 2 measurements.

There were 33 patients in the Completers analysis set (16 azithromycin, 17 placebo). For the Completers analysis there were 124 FEV_1_ measures; 26 patients with 4 measurements, 6 patients with 3 and 1 patient with 2 measurements.

The ‘as treated’ analysis used data on all 46 ITT patients and all their 177 FEV_1_ measurements. For the five placebo patients who were withdrawn and placed on open label azithromycin, their measurements post withdrawal (nine measurements in total across the five patients) contributed to the overall azithromycin treatment mean and not the placebo treatment mean.

Baseline characteristics of the study patients, for the ITT and Completer analysis sets, are summarised in table 1. All patients initially received standard immunosuppressant comprising ciclosporin, prednisolone and azathioprine. Patients with more than one episode of vascular rejection requiring augmented steroids in the 1st year post transplant and women with problematic hirsute were switched to tacrolimus treatment. In the ITT population 38 of 46 had switched to tacrolimus by enrolment; 18 in the azithromycin group and 20 in the placebo group. All switches were in the 1st year post transplant, well before study enrolment. All patients received proton pump inhibitors and statin therapy, throughout the study. There were no relevant differences in background immunosuppressant or other therapies between the two groups. Results of lavage microbiology did not lead to any change in baseline therapy and no patient enrolled was regarded as having a new infection.

**Table 1:** Baseline characteristics for the intention-to-treat (ITT, n=46) and Completer (Comp, n=33) populations, by treatment allocation group

Baseline characteristic	ITT azithromycin n=23	ITT placebo n=23	Comp azithromycin n=16	Comp placebo n=17
Sex				
Female	12 (52%)	8 (35%)	9 (56%)	7 (41%)
Male	11 (48%)	15 (65%)	7 (44%)	10 (59%)
Age (years), median (IQR)	51.0 (35–56)	51.0 (44–59)	53.5 (47.0–57.5)	54.0 (45.0–62.0)
Pretransplant (Tx) disease				
Cystic fibrosis	7 (30%)	6 (26%)	2 (13%)	4 (24%)
Emphysema	11 (48%)	8 (35%)	9 (56%)	6 (35%)
Fibrosing alveolitis	2 (9%)	4 (17%)	2 (13%)	3 (18%)
Other*	3 (13%)	5 (22%)	3 (19%)	4 (24%)
Tx procedure				
Double lung	14 (61%)	13 (57%)	7 (44%)	9 (53%)
Single lung	9 (39%)	9 (39%)	9 (56%)	7 (41%)
Heart lung	0	1 (4%)	0	1 (6%)
Years between Tx and BOS				
Median (IQR)	3.7 (1.3–7.4)	2.2 (1.3–5.0)†	4.2 (2.6–7.8)	2.0 (1.3–4.0)
BOS stage				
1	13 (57%)	17 (74%)	10 (63%)	13 (77%)
2	8 (35%)	4 (17%)	6 (38%)	3 (18%)
3	2 (9%)	2 (9%)	0	1 (6%)
FEV_1_ (litres), median (IQR)	1.5 (1.2–2.4)	1.7 (1.5–2.5)	1.6 (1.2–2.4)	1.7 (1.5–2.2)
FVC (L), median (IQR)	3.0 (2.3–3.6)	2.9 (2.2–3.6)	2.7 (2.1–3.6)	2.9 (2.1–3.5)
TBB A and B scores‡				
Missing	2 (9%)	4 (17%)	2 (13%)	3 (18%)
Ax	5 (22%)	8 (35%)	3 (19%)	7 (41%)
A0	12 (52%)	7 (30%)	7 (44%)	4 (24%)
A1	4 (17%)	3 (13%)	4 (25%)	2 (12%)
A2	0	1 (4%)	0	1 (6%)
Missing	2 (9%)	4 (17%)	2 (13%)	3 (18%)
Bx	5 (22%)	7 (30%)	5 (31%)	4 (24%)
B0	5 (22%)	6 (26%)	2 (13%)	5 (29%)
B1R	9 (39%)	6 (26%)	7 (44%)	5 (29%)
B2R	2 (9%)	0	0	0
BAL microbiology				
Missing	2 (9%)	2 (9%)	2 (13%)	1 (6%)
NPI	11 (48%)	14 (61%)	8 (50%)	11 (65%)
‘Any’ organism	10 (43%)	7 (30%)	6 (38%)	5 (29%)
‘Any’ includes: Pa	5	4	3	3
Asp Fum	2	0	1	0
Ca	4	2	2	1
Other	2	1	1	1

*Other Pre Tx disease: Obliterative Bronchiolitis, *Sarcoid*, *Congenital heart disease*, *Histiocytosis X*, *Silicosis.*

†One patient randomised to the placebo arm >10 years post transplant (at 11.9 years).

‡ISHLT grades (ref 20) BAL Microbiology=Clinical microbiology. Other=Proteus mirabilis, Stenotrophomonas Maltophilia, Ralstonia Picketti, Candida species. Percentages for patients growing individual organisms are not given since some patients grew more than one organism.

Asp Fum, Aspergillus fumigatus; BAL, bronchoalveolar lavage; BOS, bronchiolitis obliterans syndrome; Ca, Candida albicans; NPI, no pathogens identified; Pa, Pseudomanas aeruginosa; TBB, transbronchial biopsy.

### Analysis of FEV_1_ data

**Figure 2:**
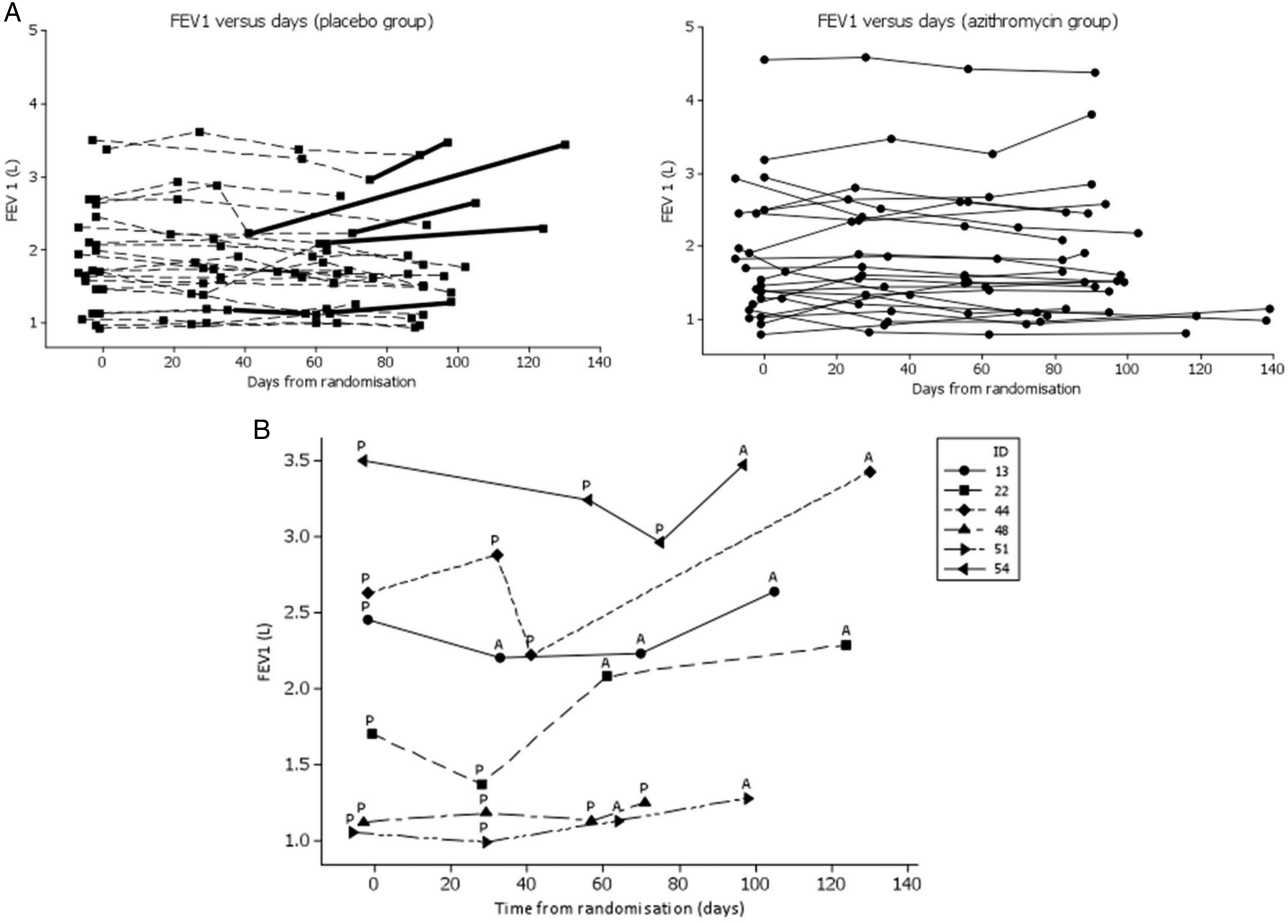
(A) FEV_1_ measurements as a two-panel spaghetti plot of FEV_1_ over time in the study. The thickened lines denote FEV_1_ from the time a patient withdrew or was withdrawn from study medication. FEV1.0 versus days (placebo group; solid squares) and FEV1.0 versus days (azithromycin group; solid circles). (B) Descriptive plot of FEV_1_ data for patients treated with placebo who withdrew or were withdrawn from study medication. Symbols, different for each patient (key) denote FEV_1_ measurements. ID, anonymised patient identifier. ‘P’ indicates where a patient was being treated with placebo at the time FEV_1_ was measured. ‘A’ denotes where a patient was being treated with azithromycin at the time FEV_1_ was measured, after withdrawal from study. Patient 48 was withdrawn from study medication following stomach pains and did not receive azithromycin. Patient 51 withdrew consent and was treated with open-label azithromycin. The remaining four patients had ‘rapid fall’ in FEV_1_ and were withdrawn and treated with open label azithromycin.

Figure 2A summarises FEV_1_ measurements as a two-panel spaghetti plot of FEV_1_ over time in the study. Figure 2B summarises FEV_1_ measurements in patients who were randomised to the placebo arm and withdrew or were withdrawn and then received open-label azithromycin. Results are summarised in table 2. For the ITT analysis (n=46, 177 observations), the estimated mean difference in FEV_1_ between treatment groups (azithromycin minus placebo) was 0.035 L, (on average higher in the azithromycin group) with a 95% CI for the mean difference of −0.112 L to 0.182 L (p=0.6). Nine out of 23 (39%) ITT patients in the azithromycin group had ≥10% gain in FEV_1_ from baseline. No patients in the placebo arm had ≥10% gain in FEV_1_ from baseline while on placebo (p<0.002, Fisher's exact test).

**Table 2:** Mean difference in FEV_1_ between treatment groups for the intention-to-treat (ITT, n=46), ‘as treated’ (n=46) and Completer (n=33) populations

Outcome FEV_1_ (L)	Mean difference in FEV_1_ (azithromycin minus placebo)	95% CI for population mean difference	p Value
	ITT analysis 46 patients, 177 measurements		
Mean difference in FEV_1_ between treatment arms, adjusted for baseline FEV_1_, randomisation stratification variables (disease and transplant) and time since randomisation	0.035	−0.112 to 0.182	0.6
	‘As treated’ analysis 46 patients, 177 measurements		
Mean difference in FEV_1_ between treatment arms, adjusted for baseline FEV_1_, randomisation stratification variables (disease and transplant) and time since randomisation	0.306	0.181 to 0.431	<0.001
	Completers analysis 33 patients, 124 measurements		
Mean difference in FEV_1_ between treatment arms, adjusted for baseline FEV_1_, randomisation stratification variables (disease and transplant) and time since randomisation	0.278	0.170 to 0.386	<0.001

For the ‘as treated’ analysis (n=46, 177 observations) the estimated mean difference in FEV_1_ between treatment groups (azithromycin minus placebo) was 0.306 L, with 95% CI for the mean difference: 0.181 L to 0.431 L (p=<0.001).

For study Completers the estimated mean difference in FEV_1_ between treatment groups (azithromycin minus placebo) was 0.278 mL, with 95% CI for the mean difference: 0.170 L to 0.386 L (p=<0.001).

### Analysis of FVC data

The results are summarised in table 3. For the ITT population, (n=46, 177 observations), the estimated mean difference in FVC between treatment groups (azithromycin minus placebo) was 0.099 L, with 95% CI for the mean difference: −0.026 L to 0.224 L (p=0.1).

**Table 3:** Mean difference in FVC between treatment groups for the intention-to-treat (ITT, n=46), ‘as treated’ (n=46) and Completer (n=33) populations

Outcome FVC (L)	Mean difference in FVC (azithromycin minus placebo)	95% CI for population mean difference	p Value
	ITT analysis 46 patients, 177 measurements		
Mean difference in FVC between treatment arms, adjusted for baseline FVC, randomisation stratification variables (disease and transplant) and time since randomisation	0.099	−0.026 to 0.224	0.1
	‘As treated’ analysis 46 patients, 177 measurements		
Mean difference in FVC between treatment arms, adjusted for baseline FVC, randomisation stratification variables (disease and transplant) and time since randomisation	0.272	0.158 to 0.386	<0.001
	Completers analysis 33 patients, 124 measurements		
Mean difference in FVC between treatment arms, adjusted for baseline FVC, randomisation stratification variables (disease and transplant) and time since randomisation	0.248	0.115 to 0.381	<0.001

For the ‘as treated’ analysis (n=46, 177 observations) the estimated mean difference in FVC between treatment groups (azithromycin minus placebo) was 0.272 L, with 95% CI for the mean difference: 0.158 L to 0.386 L (p=<0.001).

For study Completers, the estimated mean difference in FVC between treatment arms (azithromycin minus placebo) was 0.248 L, with 95% CI for the mean difference: 0.115 L to 0.381 L (p=<0.001).

### BAL neutrophil data

BAL data was not available from all patients due to a clinical decision that the sample was either not possible or prudent because of the clinical status of the patients during the bronchoscopy.

At baseline BAL differential data were available for 39/46 (85%) of the ITT analysis set. The median per cent neutrophils in BAL was 25.8% (IQR 3.4–72.0%).

**Figure 3:**
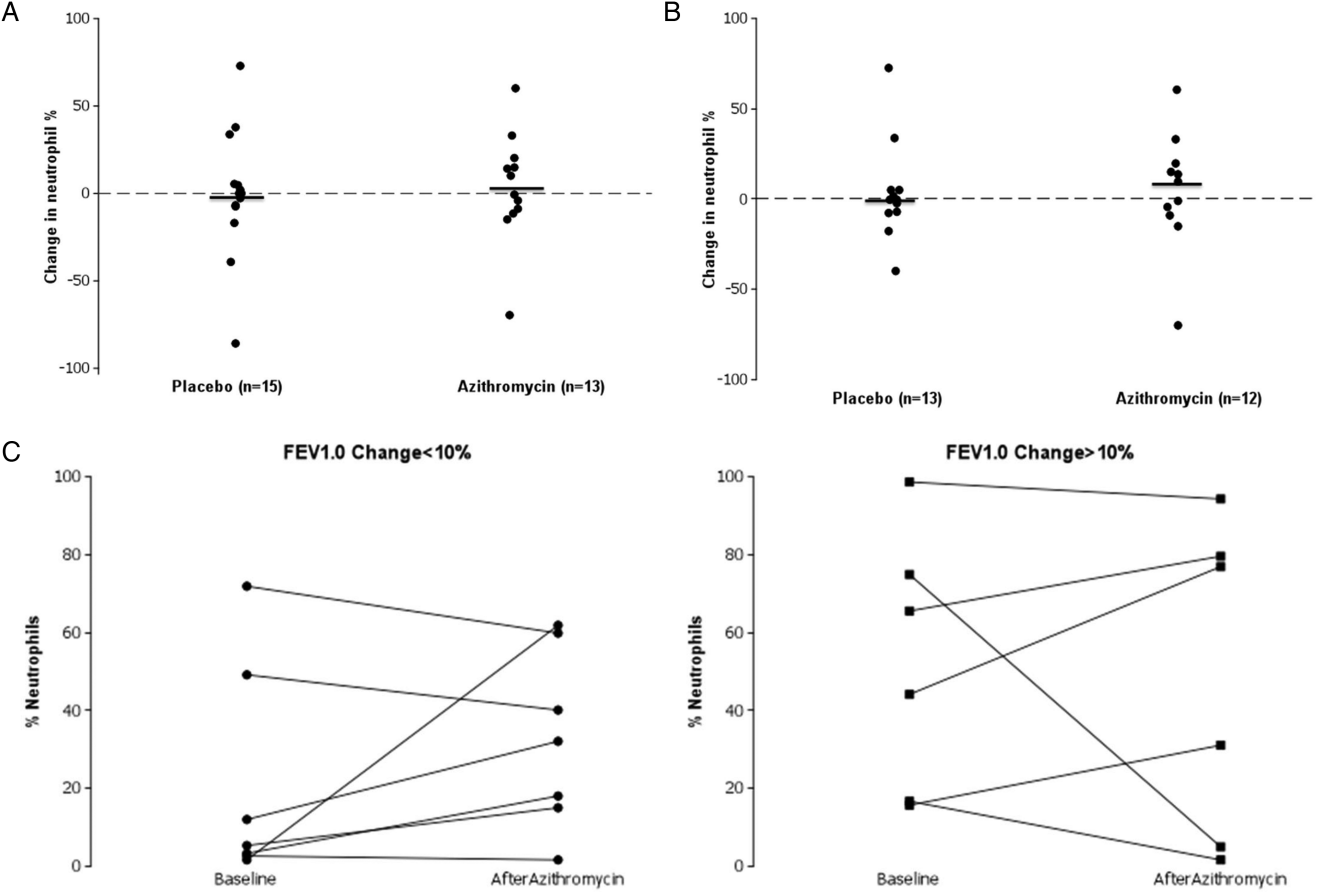
(A) The change in per cent neutrophils in bronchoalveolar lavage (BAL) from baseline to week 12 for the intention-to-treat (ITT) population, by treatment allocation group (n=28/46). Median change denoted by horizontal line. (B) The change in per cent neutrophils in BAL from baseline to week 12 for the Completer population, by treatment allocation group (n=25/33). Median change denoted by horizontal line. (C) The change in per cent neutrophils in baseline to week 12 for patients treated with azithromycin who had <10% gain in FEV_1_ (solid circles) and in patients treated with azithromycin who had a >10% gain in FEV_1_ (solid squares).

BAL neutrophil data were available at baseline and final visit for 28/46 patients in the ITT analysis set (13/23 azithromycin, 15/23 placebo) and 25/33 in the Completers analysis set (12/16 azithromycin, 13/17 placebo). Summary statistics for baseline, final and change in BAL neutrophil percentage are given in table 4. There was no evidence of systematic changes in BAL neutrophil percentage associated with either azithromycin or placebo treatment for either the ITT (figure 3A) or study Completer populations (figure 3B).

**Table 4:** Per cent neutrophils in bronchoalveolar lavage at baseline and final visit (week 12) for the intention-to-treat (ITT) (n=28/46) and Completer (n=25/33) populations, by treatment allocation

	n	Baseline Median (IQR)	Final visit Median (IQR)	Change from baseline Median (IQR)
ITT azithromycin	13/23	16.6 (4.2 to 68.8)	32.0 (10.0 to 69.5)	9.8 (−10.4 to 17.7)
ITT placebo	15/23	14.8 (2.4 to 56.0)	19.8 (2.0 to 52.2)	−0.5 (−7.8 to 5.0)
Completers azithromycin	12/16	16.1 (3.7 to 61.5)	31.5 (7.5 to 73.3)	11.9 (−7.7 to 18.9)
Completers placebo	13/17	9.2 (2.0 to 52.5)	19.8 (1.5 to 46.9)	−0.5 (−7.5 to 4.9)

### Transbronchial biopsy data

Where paired data were available approximately half of the biopsies were graded as ‘Bx’ (ungradeable) for the B scores and a third were ‘Ax’ (ungradeable) for the A scores. Further analysis of the potential effect of azithromycin on biopsy scores was therefore not carried out. The data is summarised in online supplementary appendix 3.

### Safety

Seven adverse events led to hospital admission or the prolonging of existing hospitalisation. They were therefore classified as serious, but not related to study medication. There were no other major safety issues to report with the trial.

## Discussion

To our knowledge this is the first randomised controlled study of azithromycin therapy in BOS. Our trial showed that azithromycin improves FEV_1_ and FVC in a significant proportion of patients. As in previous open studies not all patients with BOS responded to azithromycin.

We also noted that in patients treated with placebo who were withdrawn from the study due to rapid fall in lung function, treatment with open label azithromycin was associated with a significant gain in lung function leading to our decision to report our Completers and ‘as treated’ groups. We recognise that our ‘as treated’ analysis should be interpreted cautiously, as a post hoc analysis. Overall we conclude that azithromycin appears superior to placebo treatment in our study.

An implication of the present study is that the definition of BOS, which currently includes the presence of irreversible airflow obstruction, should be revised or a new term introduced to describe a phenotype of patient who fulfils the definition of BOS, apart from showing a response to azithromycin. This has been suggested prominently by others.^5^

A limitation of our study was randomisation of 48 subjects versus the target of 64. The incidence of BOS throughout the potential trial population was below that estimated. Consequently, our recruitment rate was on average one per month rather than the estimated rate of two per month. Our recruitment period ran for 48 months rather than the anticipated 30 months. This resulted in having data available from 46 patients for the ITT analysis compared with the target of 58. This shortfall would be expected to reduce the power of the study. However, the observed SD of the change in FEV_1_ from baseline was also much smaller than anticipated at design, a factor that would be expected to favour the study power. In line with CONSORT guidelines,^25^ we reported 95% CIs for the trial, allowing open interpretation of the findings to be considered along with study size.

Our study compliments a previous randomised trial in lung transplantation where azithromycin was used as prophylaxis against developing BOS. This had a primary end point of freedom from BOS and survival 2 years after lung transplantation.^26^ This found that BOS-free survival was better with azithromycin. Patients receiving azithromycin had better FEV_1_, and lower airway neutrophilia.^26^ When open-label azithromycin was initiated in patients with BOS, this was associated with an improvement of FEV_1_ in around half the patients treated.^26^

Our study was powered to detect a change in FEV_1_. FEV_1_ is the basis for the internationally recognised classification of BOS and has previously been reported in open studies of azithromycin therapy,^9–13^ and was reported in the sole previous randomised trial of azithromycin for BOS prophylaxis.^26^FVC may be sensitive to changes in the calibre of smaller, peripheral airways.^27^ ^28^ Our data indicated that azithromycin was superior to placebo for FVC in the Completer population and ‘as treated’ analysis.

Our current and previously reported data^29^ therefore suggest that FVC and other tests such as FEF_25–75%_ may be useful end points for intervention studies in BOS. Previous open studies of azithromycin therapy, including work from our own centre have not reported FVC data^9–13^ or focused on physiological measurements which reflect smaller airway function. It would seem reasonable to report such measurements given the recognised small airway contribution to BOS pathophysiology, and that the measurements are readily made.

As a secondary end point we analysed the effects of azithromycin therapy on BAL neutrophils, reflecting our long-standing interest^30^ ^31^ BOS has a neutrophilic pathophysiology and it has been suggested that the clinical heterogeneity of BOS may be clarified by considering a distinct patient subset with neutrophilic reversible allograft dysfunction.^5^ This proposed dichotomy may have important therapeutic implications in predicting patients who might respond to azithromycin.^5^ In a significant number of our patients no BAL data were available. The samples that were available confirmed our previous finding,^30^ and others’^32^ ^33^ that BOS is accompanied by an elevated BAL neutrophil count. However azithromycin therapy was not associated with a change in neutrophils. Our findings are therefore different to the Leuven Centre which has published data indicating that a fall in neutrophilic inflammation occurs in patients with a clinical response to azithromycin. We feel that the limited data available for analysis in our study precludes firm conclusions being drawn. We would therefore recommend that further research is performed to clarify the relationship between BAL neutrophil levels and the clinical effectiveness of azithromycin treatment.

Apart from potential anti-inflammatory benefits, which may include effects on neutrophil numbers and function, other effects of macrolide therapy have been reviewed elsewhere and warrant further study. These include immunomodulatory mechanisms, interference in the formation of infective biofilms and alleviation of extraoesophageal reflux and microaspiration, with promotion of gastric motility.^34^ It is increasingly recognised that reflux and aspiration may be an important injury in lung allografts,^35^ and we would recommend that characterisation of reflux disease is considered in future intervention trials in BOS.

In our study azithromycin treatment was not associated with significant adverse events. There were no deaths or graft losses during the study. It has been suggested that prolonged treatment with azithromycin may have adverse effects which may be of potential relevance in lung transplantation. These include gastrointestinal effects, loss of hearing^36^ and the development of macrolide resistant organisms.^34^ It is also suggested that azithromycin could predispose patients to the development of non-TB mycobacterial infection.^37^ Debate has also been generated by a study of azithromycin use in a group of patients with pre-existing heart disease. Here azithromycin treatment was associated with an increased rate of cardiovascular related mortality.^38^ It has also been shown that the macrolide antibiotics may cause cholestatic hepatitis at an estimated rate of 3.6 per 100 000.^39^

While azithromycin treatment in BOS is generally safe, and provides a therapeutic opportunity in a pathophysiology causing significant morbidity and mortality,^9^ ^10^ ^12–16^ ^26^ ^40^ it remains a research priority to elucidate which patients benefit from azithromycin, what the optimum timing of treatment is and to provide long-term follow-up data. This might lessen the possibility of iatrogenic consequences of therapy, although these are also the subject of investigation and debate, with the possibility that risks have been overestimated generally, and may not be especially relevant in the specialised setting of lung transplantation. We consider the potential benefits of alternate day low dose azithromycin 250 mg outweigh the potential risks in lung transplantation. Ideally the results of this trial should be replicated.

We conclude that this study provides strengthened evidence for the clinical practice of initiating azithromycin therapy for patients who develop BOS post lung transplantation.

## Supplementary Material

Web supplement
