# Supplementary material for: A randomised controlled trial of azithromycin therapy in bronchiolitis obliterans syndrome (BOS) post lung transplantation
Source: Thorax. 2015 Feb 24;70(5):442–50. doi: 10.1136/thoraxjnl-2014-205998 (PMC4413845; doi:10.1136/thoraxjnl-2014-205998)
Supplement: Web supplement [file thoraxjnl-2014-205998-s1.pdf]

Appendix 1: Results from the analysis of baseline and week 12 data

Table 1: Change in FEV1.0 for the ‘true’ intention to treat population (ITT, *n*=46)

Outcome<br><br>Change in FEV_1 (litres) from baseline	ITT, n=46				
	Azithromycin mean change from baseline n=23	Placebo mean change from baseline n=23	Mean difference in change (Azith minus Placebo)	95% CI for population mean difference in the change	P-value
Absolute mean change* in FEV_1 from baseline to final, adjusted for baseline FEV_1 and randomisation stratification variables (disease and transplant)	− 0.021	− 0.040	0.019	-0.210 to 0.247	P=0.9

* A negative mean change indicates a fall on average in FEV_1 from baseline

Table 2: Change in FEV1.0 for the modified ‘intention to treat’ population utilising lung function at the last pre-withdrawal observation for those 13 patients (7 Azithromycin, 6 Placebo) who withdrew or were withdrawn from study treatment (ITT, *n*=46)

Outcome<br><br>Change in FEV_1 (litres) from baseline	ITT, n=46				
	Azithromycin mean change from baseline n=23	Placebo mean change from baseline n=23	Mean difference in change (Azith minus Placebo)	95% CI for population mean difference in the change	P-value
Absolute mean change* in FEV_1 from baseline to final, adjusted for baseline FEV_1 and randomisation stratification variables (disease and transplant)	0.041	− 0.185	0.226	0.069 to 0.384	P=0.006

* A negative mean change indicates a fall on average in FEV_1 from baseline

Table 3: Change in FEV1.0 for the Completers (*n*=33)

Outcome<br><br>Change in FEV_1 (litres) from baseline	Completers, n=33				
	Azithromycin mean change from baseline n=16	Placebo mean change from baseline n=17	Mean difference in change (Azith minus Placebo)	95% CI for population mean difference in the change	P-value
Absolute mean change* in FEV_1 from baseline to final, adjusted for baseline FEV_1 and randomisation stratification variables (disease and transplant)	0.219	− 0.149	0.367	0.198 to 0.537	P<0.001

* A negative mean change indicates a fall on average in FEV_1 from baseline

Appendix 2

Chronological listing of patients who withdrew (2) or were withdrawn (11) from study drug. Twelve of the 13 withdrawals commenced open label azithromycin; Subject 48 was not put on open label azithromycin because withdrawal from study medication was due to stomach pains.

Subject ID	Treatment allocation	Rand date	Withdrawal date	Days in the study (12 weeks = 77 to 91 days)	Reason/comment
04	Azithromycin	10.01.2007	15.01.2007	5	Withdrew consent.
07	Azithromycin	15.03.2007	21.03.2007	6	Rapid rate of fall in FEV1.
13	Placebo	19.07.2007	07.08.2007	19	Rapid fall in FEV1.
18	Azithromycin	15.02.2008	15.03.2008	29	Increasing SOB
22	Placebo	15.05.2008	12.06.2008	28	Rapid rate of fall in FEV1.
34	Azithromycin	02.12.2008	12.02.2009	72	Increasing SOB and reduced air entry right lung
39	Azithromycin	21.05.2009	22.06.2009	32	Rapid rate of fall in FEV1
43	Azithromycin	02.09.2009	29.09.2009	27	Rapid fall in FEV1
44	Placebo	11.03.2010	21.04.2010	41	Rapid fall in FEV1.
45	Azithromycin	03.12.2009	29.12.2009	26	Rapid rate of fall in FEV1
48	Placebo	08.02.2010	09.03.2010	29	Stopped trial medication due to stomach pains.
51	Placebo	21.06.2010	20.07.2010	29	Withdrew consent.
54	Placebo	04.11.2010	18.01.2011	75	Rapid fall in FEV1.

Appendix 3: Baseline and 12 week TBB ISHLT grading scores (reference 22) at baseline and following treatment.

Of the 46 ITT patients, biopsies were assessed in 40/46 at baseline (21/23 azithromycin, 19/23 placebo) and 33/46 at final (15/23 azithromycin, 18/23 placebo). There were 29/46 patients with both baseline and final visit biopsy assessment (13/23 azithromycin, 16/26 placebo). Where paired data were available approximately half of the biopsies were graded as “Bx” (ungradeable) for the B scores and a third were “Ax” (ungradeable) for the A scores. Further analysis of the potential effect of azithromycin on biopsy scores was therefore not carried out.

Azithromycin treatment arm: Baseline and Final A scores (n=13/23)

**Baseline A Scores**	**Final A scores**				**Total**
	Ax	A0	A1	A2	
**Ax**	0	2	0	0	**2**
**A0**	0	4	2	1	**7**
**A1**	0	2	2	0	**4**
**A2**	0	0	0	0	**0**
**Total**	**0**	**8**	**4**	**1**	**13**

Placebo treatment arm: Baseline and Final A scores (n=16/23)

**Baseline A Scores**	**Final A scores**				**Total**
	Ax	A0	A1	A2	
**Ax**	2	4	1	1	**8**
**A0**	0	4	0	1	**5**
**A1**	0	1	0	1	**2**
**A2**	0	1	0	0	**1**
**Total**	**2**	**10**	**1**	**3**	**16**

Azithromycin treatment arm: Baseline and Final B scores (n=13/23)

**Baseline B Scores**	**Final B scores**				**Total**
	Bx	B0	B1	B2	
**Bx**	3	1	1	0	**5**
**B0**	0	1	1	0	**2**
**B1**	1	1	4	0	**6**
**B2**	0	0	0	0	**0**
**Total**	**4**	**3**	**6**	**0**	**13**

Placebo treatment arm: Baseline and Final B scores (n=16/23)

**Baseline B Scores**	**Final B scores**				**Total**
	Bx	B0	B1	B2	
**Bx**	3	2	0	0	**5**
**B0**	1	3	1	0	**5**
**B1**	3	1	2	0	**6**
**B2**	0	0	0	0	**0**
**Total**	**7**	**6**	**3**	**0**	**16**
